# The Pathogenic Role of Oxidative Stress, Cytokine Expression, and Impaired Hematological Indices in Diabetic Cardiovascular Diseases

**DOI:** 10.1007/s10753-022-01718-w

**Published:** 2022-08-23

**Authors:** Howaida Saad, Hanan A. Soliman, Basant Mahmoud, Adel Abdel Moneim, Mohamed Y. Zaky

**Affiliations:** 1 Department of Biochemistry Faculty of Science, Beni-Suef University, Beni-Suef, Egypt; 2 Molecular Physiology Division, Faculty of Science, Beni-Suef University, Salah Salem St, Beni-Suef, 62511 Egypt

**Keywords:** cardiovascular diseases, diabetes, myocardial infarction, heart failure, oxidative stress, cytokines, hematological indices.

## Abstract

A simultaneous increase in the prevalence of diabetes mellitus (DM), a risk factor for cardiovascular diseases (CVDs), has contributed to the escalation of CVD related mortalities. To date, oxidative stress and inflammation are increasingly recognized as significant drivers of cardiovascular complications in patients with diabetes. Therefore, this study aims to explore the correlation between oxidative stress, inflammation, and hematological indices in diabetic patients with CVDs. Patients were allocated into five groups: healthy controls; nondiabetic patients with myocardial infarction; diabetic patients with myocardial infarction; nondiabetic patients with heart failure; and diabetic patients with heart failure. The results revealed that the malondialdehyde levels were increased; whereas superoxide dismutase enzyme activities were markedly reduced in all CVD groups compared with those of healthy controls. Although the mRNA expression levels of interleukin (IL)-6, IL-18, and IL-38 were significantly increased, those of the anti-inflammatory cytokine, IL-35, have been reduced in all CVD groups compared with healthy controls. Regarding hematological indices, hematocrit, red blood cell distribution width, mean platelet (PLT) volume, plateletcrit, PLT distribution width, leukocyte count, and PLT-to-lymphocyte and neutrophil-to-lymphocyte ratios were markedly increased in the diabetic and nondiabetic CVD groups compared with those of the healthy controls. Oxidative stress and cytokine biomarkers may play a significant role in the complications of diabetic cardiomyopathy. Moreover, hematological indices are particularly sensitive to systemic inflammatory changes and are novel markers for the early detection of diabetic cardiomyopathy.

## INTRODUCTION

Diabetes mellitus (DM) is a global health issue and is concomitant with cellular, metabolic, and blood cell abnormalities [1]. The American Heart Association regards diabetes as one of the key risk factors for cardiovascular diseases (CVDs) [2]. DM is often accompanied by cardiovascular disturbances, including coronary artery disease (CAD) and peripheral artery disease. Diabetes-induced pathological disorders may cause tissue damage in one-third to one-half of people with diabetes [3].

Oxidative stress is an imbalance in reactive oxygen species (ROS) and antioxidant defense mechanisms [4]. Cardiovascular insulin resistance, diabetic cardiomyopathy, and heart failure are accelerated by oxidative stress [5]. Recently, hypertriglyceridemia was demonstrated to increase CVD-related deaths in patients with diabetes [6]. According to human and animal studies, hyperglycemia may trigger an inflammatory response through oxidative pathways, which results in cardiac fibrosis and cardiomyocyte death, followed by cardiac dysfunction [7]. Indeed, inflammation, which is common in diabetes, can impact cardiomyocyte contractility and survival through various mechanisms, such as peroxynitrite formation and alterations in the extracellular matrix composition; dynamics are hypothesized to contribute to cytokine-induced cardiac contractile failure [8]. Interleukin (IL)-6 plays a vital role in the pathophysiology of CVD and its complications. Different immune responses and susceptibility to CAD are produced by genetic differences in IL-6 [9]. IL-38, a recently revealed member of the IL-1 cytokine family with anti-inflammatory activity, is generally recognized as a key regulator of inflammation. IL-38 is commonly expressed in the heart, and IL-38 polymorphisms are associated with coronary artery syndrome [10]. Moreover, IL-35, an anti-inflammatory cytokine, is involved in various disorders, including CVD and diabetes [11]. Hematological alterations in diabetes may occur due to ROS production as a consequence of long-term hyperglycemia [12]. Excessive ROS production causes oxidative stress, which leads to tissue damage and hematological alterations, including endothelial dysfunction, platelet (PLT) hyperactivity, and red blood cell (RBC) dysfunction [13]. Hematological alterations have been encountered in patients with DM, including alterations in the structure, function, and metabolism of PLT, RBC, white blood cell (WBC), and the coagulation system [12].

The role of inflammation in diabetic CVD has been recently reported; however, its mechanism requires more exploration. In particular, the interplay between DM-induced inflammation and hematological disorders in the risk of CVD requires further investigation. Therefore, this study aims to explore the relationship between oxidative stress, inflammation, and alteration in hematological indices and the risk of developing diabetic cardiomyopathy.

## MATERIALS AND METHODS

### Study Population

A total of 120 patients with both myocardial infarction (diabetic type 2 and nondiabetic) and heart failure (diabetic type 2 and nondiabetic) who visited the Cardiac and Chest Intensive Care Unit of Beni-Suef University Hospital (Beni-Suef, Egypt) were enrolled in this cross-sectional study. The data was collected using a paper questionnaire and clinical laboratory reports. Eligible patients were allocated into four groups based on the clinical and biochemical investigations. Moreover, thirty normal healthy participants were selected as healthy controls. Moreover, all patients provided a written consent and blood samples during the period from March 2019 to November 2019 following protocol approval by the Beni-Suef University Hospital Ethics Committee (BNS/2019/2).

### Inclusion and Exclusion Criteria and Experimental Design

**Fig. 1:**
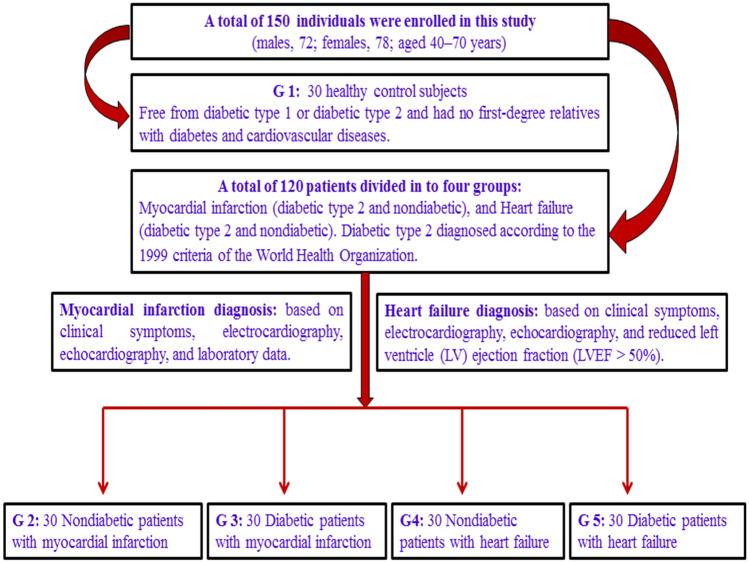
Schematic diagram presenting inclusion and exclusion criteria and experimental design.

The adult patients (males, 72; females, 78; aged 40–70 years), including healthy controls, diabetic and nondiabetic patients with heart failure, and diabetic and nondiabetic patients with myocardial infarction, were enrolled in this study (Fig. 1). The healthy subjects consisted of participants who had no significant health-related issues. All participants (healthy controls and patients) were free of asthma symptoms, alcohol abuse, infectious diseases, allergies, kidney failure, eczema, thyroid diseases, autoimmune disorders, and liver dysfunction.

### Biochemical Investigations

After overnight fasting, blood samples were collected in sodium fluoride, ethylenediaminetetraacetic acid (EDTA), and plain tubes (5 mL each). The EDTA blood samples were used to estimate complete blood count and glycated hemoglobin (HbA1c). Samples were stored at − 40 °C until use. Sodium fluoride tubes were used to collect blood samples to determine fasting blood sugar (FBS) level. Plasma FBS and serum cholesterol, high-density lipoprotein (HDL), and triglycerides levels were assessed using a reagent kit (Spinreact Co., Spain). Subsequently, low-density lipoprotein (LDL) and very low-density lipoprotein (vLDL) levels were calculated based on the formula of Friedewald *et al*. [14]. Cardiovascular risks (1 and 2) were calculated following the formula of Ross [15]. The HbA1c level was determined using a reagent kit (Stanbio Laboratory, TX, USA). Furthermore, serum insulin was assessed using radioimmunoassay kits (Diagnostic Products Corporation, LA, USA). Homeostatic model assessment for insulin resistance (HOMA-IR) values was calculated using the following equation: HOMA-IR = fasting insulin (U/L) × fasting glucose (mg/dL)/405 [16]. The C-reactive protein (CRP) was estimated using a reagent kit (Spinreact Co., Spain). Serum CK-MB and troponin I levels were determined using test kits (HUMAN, Germany). Moreover, malondialdehyde (MDA) levels and superoxide dismutase (SOD) activities were determined using a reagent kit (BioVision, Milpitas, CA, USA). Hematology parameters, including hemoglobin, RBCs, hematocrit, mean corpuscular hemoglobin, mean corpuscular hemoglobin content, mean corpuscular volume, RBC distribution width (RDW), PLT count, plateletcrit (PCT), PLT distribution width (PDW), mean PLT volume (MPV), WBC count, and differential leukocyte count, were determined using a MICROS ABX autoanalyzer. All procedures were performed following the kit manufacturers’ instructions.

### Real-Time Polymerase Chain Reaction

RNA was isolated from the blood samples using a GeneJET™ RNA purification kit (Thermo Fisher Scientific Inc., Rochester, NY, USA). RNA was purified and spectrophotometrically quantified. Consequently, the target DNA cDNA was amplified using GoTaq Green Master Mix (Promega, WI, USA) using the following sets of primers: 5ˋ-GGTACATCCTCGACGGCATCT-3ˋ (forward primer) and 5ˋ-GT GCCTCTTTGCTGCTTTCAC-3ˋ (reverse primer) for IL-6, 5ˋ -GCTTCCTCTCGC AAC AAA C-3ˋ(forward primer) and 5ˋ -CACTTCACAGAGATAGTTACAGCC-3ˋ(reverse primer) for IL-18, 5ˋ-TGTTCTCCATGGCTCCCTA-3ˋ(forward primer) and 5ˋ-TTATGAAAGGCACGAAGCTG-3ˋ(reverse primer) for IL-35, 5ˋ- AAGAAGGACCTCCGGCTCT -3ˋ(forward primer) and 5ˋTGACTCAGAATCTGGC5GTATTTC-3ˋ (reverse primer) for IL-38, and 5ˋ- TCACCCTGAAGTACCCCATGGAG-3ˋ(forward primer) and 5ˋ TTGGCCTTGGGGTTCAGGGGG -3ˋ (reverse primer) for β-actin. Green Master Mix (Promega, WI, USA) and T100TM thermal cycler (Bio-Rad Laboratories, CA, USA) were used for PCR under the following conditions: initial denaturation at 95 °C for 5 min, 35 cycles set at 95 °C (1 min) for denaturation, 60 °C (1 min) for annealing, and 72 °C (1 min) for extension, and ultimately at 72 °C (5 min) to finish the extension reaction. Values were normalized to the quantity of β-actin. All molecular assays were conducted at the Molecular Biology Laboratory of CliniLab (Cairo, Egypt).

### Statistical Analysis

Results were presented as mean values and SEM. Shapiro–Wilk normality test showed that the data were not normally distributed, and thus a non-parametric Kruskal–Wallis test followed by the post hoc test using a pairwise multiple-comparative analysis was used to determine the statistical differences between groups using a computer software package (SPSS version 20, IBM Corp., 2011). A simple linear correlation study was conducted using Spearman’s correlation analysis to estimate the degree of the relationship between the variables. A *P* value < 0.05 was considered statistically significant.

## RESULTS

The results revealed that the body mass index values were significantly higher (*p* < 0.001) in all CVD patient groups compared with the healthy control group. Meanwhile, all patient groups showed a significant (*p* < 0.001) increase in systolic and diastolic blood pressures compared with the healthy control group. Moreover, the diabetic myocardial infarction and diabetic heart failure groups showed a significant (*p* < 0.001) increase in the FBS level compared with the nondiabetic myocardial infarction, nondiabetic heart failure, and healthy control groups. However, the diabetic CVD groups exhibited a significant (*p* < 0.001) decline in the insulin level compared with the nondiabetic CVD and healthy control groups. The diabetic myocardial infarction and diabetic heart failure groups revealed a significant (*p* < 0.001) increase in HbA1c% and HOMA-IR values compared with the nondiabetic CVD and healthy control groups. All CVD patient groups showed a highly significant (*p* < 0.001) increase in serum CK-MB and troponin I levels compared with the healthy control group, with a marked increase in both myocardial infarction groups (Table 1).

**Table 1:** Demographic and Biochemical Characteristics of Healthy Controls and Diabetic and Nondiabetic CVD Dysfunction Groups

Age	48.7±4.0	61.2±10.8***	59.2±10.5***	59.0±11.2***	65.3±10.0***
Gender	M 22 (73.3%)	M 20 (66.7%)	M 22 (73.3%)	M 20 (66.7%)	M 22 (73.3%)
	F 8 (26.7%)	F 10 (33.3%)	F 8 (26.7%)	F10 (33.3%)	F 8 (26.7%)
BMI	23.8±0.6	27.9±2.2*	27.6±2.8*	28.9±2.2*	28.9±3.0*
Smoking	2 (6.7%)	18 (60%)	15 (50.0%)	14 (46.7%)	14 (46.7%)
Duration of CVD	--	3.3±1.4	4.5±1.7 N.S.	8.2±2.5	8.9 ±2.5 N.S.
Duration of DM	--	--	11.3±3.5	--	12.1±4.5
Systolic BP	105.3±6.3	154.7±12.8***	153.3±12.7***	158.0±14.9***	156.0±14.8***
Diastolic BP	75.3±5.1	98.7±8.2***	96.7±8.8***	98.7±9.0***	98.7±9.7***
FBS (mg/dl)	72.5±0.8	72.9±0.7 N.S.	197.3±7.9***###	72.9±0.7 N.S.	179.1±5.7***###
Insulin (mIU/ml)	10.1±0.1	10.0±0.1 N.S.	7.4±0.2***##	10.1±0.1 N.S.	8.6±0.2***##
HbAIC%	5.0±0.1	4.6±0.1 N.S.	9.3±0.4***###	4.7±0.1 N.S.	9.9±0.3*** ###
HOMA-IR	1.8±0.0	1.9±0.1N.S.	3.6±0.1***###	2.0±0.0 N.S.	3.7±0.0***###
Troponin I (ng/ml)	0.2±0.0	2.9±0.1***	3.1±0.1***	1.7±0.0***	1.8±0.1***
CK-MB (U/L)	11.3±0.3	66.8±2.6***	68.8±4.2***	53.2±2.7***	50.0±1.8***

Mean values and SEM are represented. A non-parametric Kruskal–Wallis test followed by the post hoc test using a pairwise multiple-comparative analysis was used to determine the statistical difference between groups, number of individuals = 150. ^**^p < 0.01 and ^***^p < 0.001 compared with the healthy control group and with ^##^*p* < 0.01 and ^###^*p* < 0.001 compared with the respective nondiabetic group

*NS* not significant, *BMI* body mass index, *BP* blood pressure, *FBS* fasting blood sugar, *HbAIc* glycosylated hemoglobin, and *CK-MB* creatine kinase myocardial band activity

**Fig. 2:**
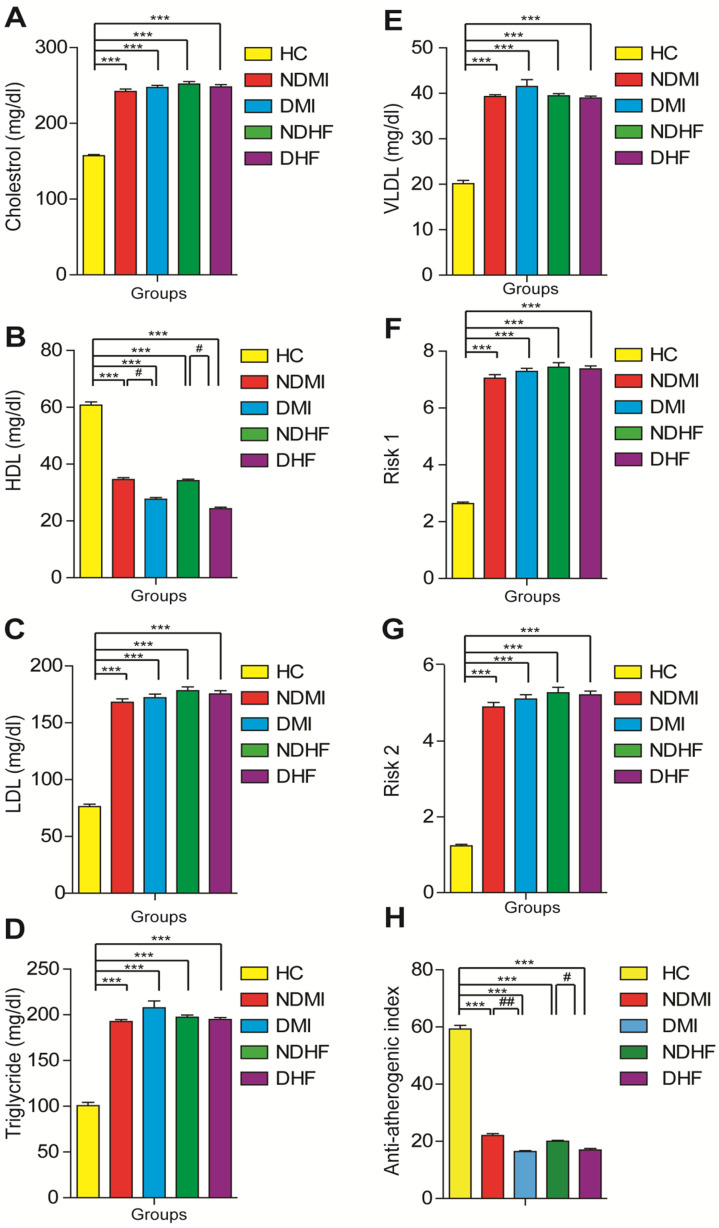
The changes in the values of** A** cholesterol, **B** HDL, **C** LDL, **D** triglycerides, **E** vLDL **F** risk factor 1, **G** risk factor 2, and **H** anti-atherogenic index among healthy controls, myocardial infarction (non-diabetic and diabetic), and heart failure (non-diabetic and diabetic) groups. Mean values and SEM are represented. A non-parametric Kruskal–Wallis test followed by the post hoc test using a pairwise multiple-comparative analysis was used to determine the statistical difference between groups, *p < 0.05, **p < 0.01, ***p < 0.001 as compared to healthy control and with ^#^*p* < 0.05, ^##^*p* < 0.01 as compared with non-diabetic groups. N.S. was not significant. LDL, low-density lipoprotein cholesterol; HDL, high-density lipoprotein cholesterol; vLDL, very low-density lipoprotein cholesterol.

Regarding lipid profile results, all CVD groups exhibited a significant (*p* < 0.001) increase in serum triglycerides, cholesterol, LDL-c, and vLDL-c levels, as well as cardiovascular risks 1 and 2 values, than healthy controls. However, HDL and anti-atherogenic index values showed a notable (*p* < 0.001) reduction in the diabetic CVD groups compared with those in the nondiabetic CVD and healthy control groups (Fig. 2).

**Fig. 3:**
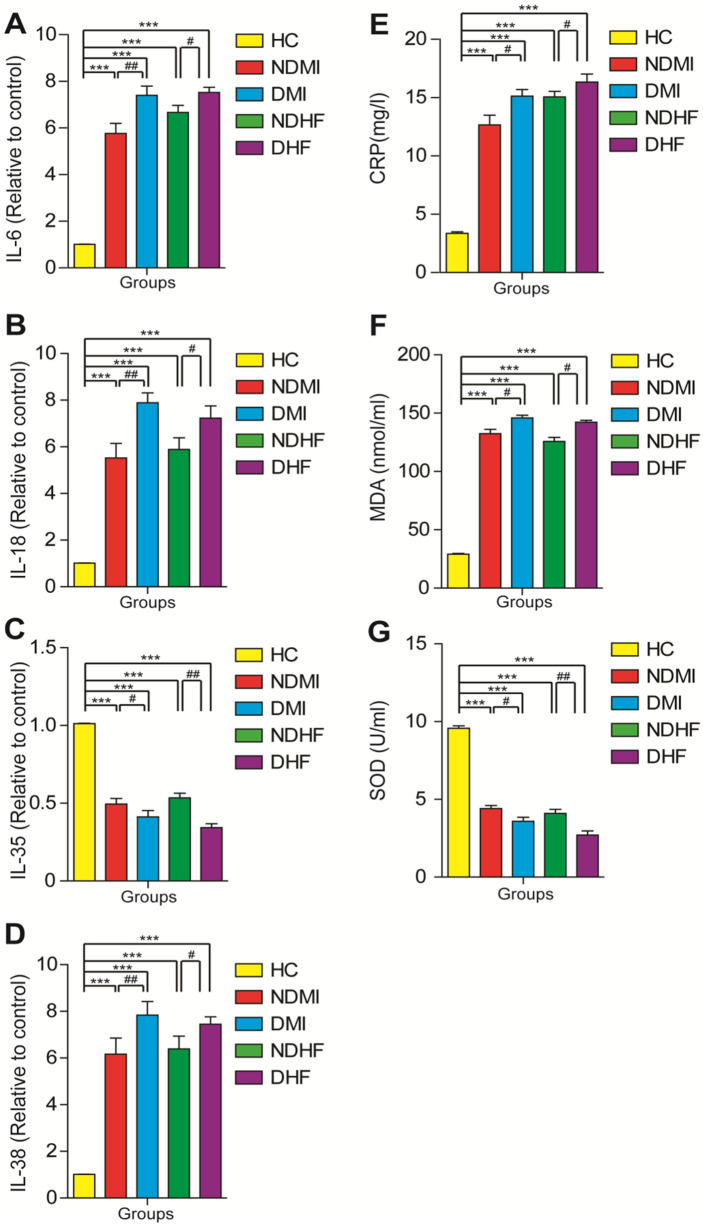
The changes in the values of **A** IL-6, **B** IL-18, **C** IL-35, **D** IL-38, **E** CRP, **F** MDA, and **G** SOD among healthy controls, myocardial infarction (non-diabetic and diabetic), and heart failure (non-diabetic and diabetic) groups. Mean values and SEM are represented. A non-parametric Kruskal–Wallis test followed by the post hoc test using a pairwise multiple-comparative analysis was used to determine the statistical difference between groups, *p < 0.05, **p < 0.01, ***p < 0.001 when compared to healthy control and with ^#^*p* < 0.05, ^##^*p* < 0.01, ^###^*p* < 0.001 when compared with non-diabetic groups. N.S. was not significant. IL, interleukin; MDA, malondialdehyde; SOD, superoxide dismutase; C-RP, C-reactive protein.

The expression of pro-inflammatory markers, IL-6, IL-18, and IL-38 showed a significant (*p* < 0.001) upregulation in CVD groups compared with those in the healthy control group and exhibited a significant increase in diabetic patients with CVD compared with that in nondiabetic CVD patients. In contrast, the diabetic and nondiabetic CVD groups showed a highly significant (*p* < 0.001) downregulation in the IL-35 mRNA expression compared with the healthy control group, with lower levels in diabetic patients than that in nondiabetic CVD patients. All CVD patient groups exhibited a highly significant (*p* < 0.001) increase in serum CRP levels compared with the healthy control group. Regarding oxidative stress markers, the CVD patient groups showed a highly significant (*p* < 0.001) increase in MDA levels and a remarkable (*p* < 0.001) decline in SOD levels compared with the healthy control group. However, the MDA levels exhibited a significant (*p* < 0.01) increase in the diabetic CVD patient groups compared with those in the healthy control group. Furthermore, SOD levels were significantly decreased (*p* < 0.05) in CVD diabetic groups than in CVD non-diabetic groups (Fig. 3).

**Fig. 4:**
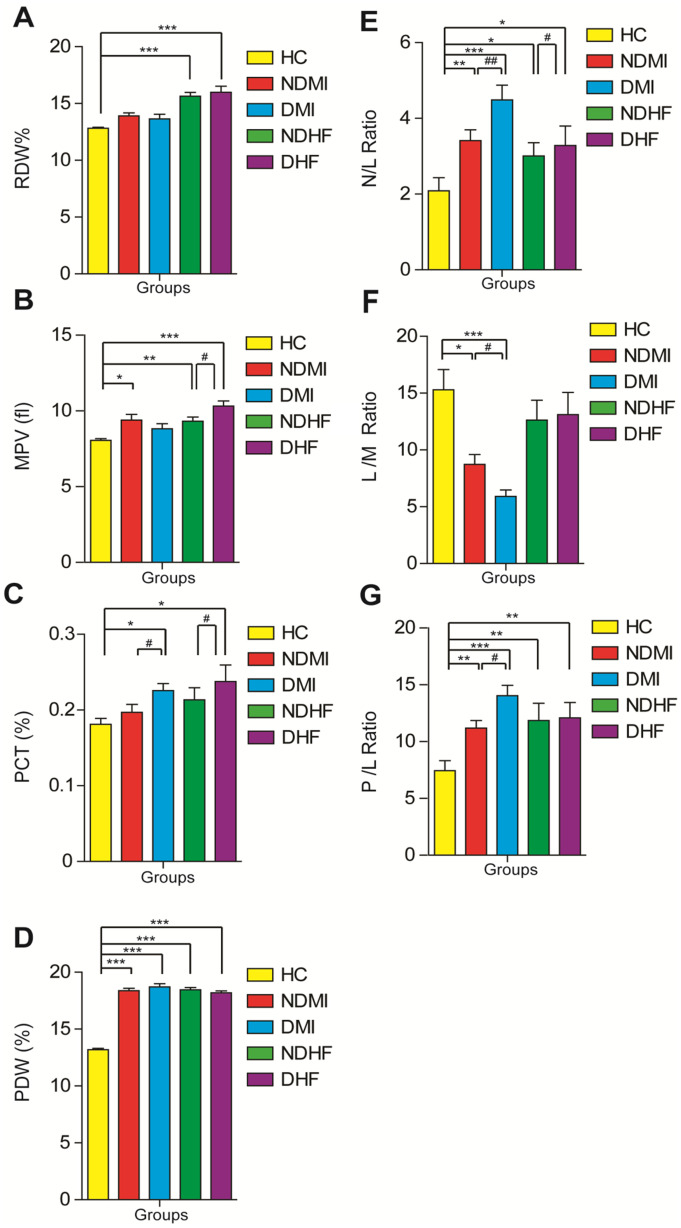
The changes in the values of** A** RDW%, **B** MPV, **C** PCT%, **D** PDW%, **E** N/L ratio, **F** L/M ratio, and **G** P/L ratio among healthy controls, myocardial infarction (non-diabetic and diabetic), and heart failure (non-diabetic and diabetic) groups. Mean values and SEM are represented. A non-parametric Kruskal–Wallis test followed by the post hoc test using a pairwise multiple-comparative analysis was used to determine the statistical difference between groups, *p < 0.05, **p < 0.01, ***p < 0.001 when compared to healthy control and with ^#^*p* < 0.05 when compared with non-diabetic groups. N.S. was not significant. PDW, platelets distribution width; RDW, red distribution width; PCT, plateletcrit; MPV, mean platelets volume; N/L, neutrophil/lymphocyte, N/L, lymphocyte/monocyte; P/L, platelets/lymphocyte.

In the present study, both heart failure patient groups exhibited a significant decrease in the hemoglobin content, RBC count, and hematocrit values compared with the healthy control group (Table 2). Moreover, the heart failure patient groups revealed a significant (*p* < 0.001) increase in RDW% compared with the healthy control group. Additionally, all CVD patient groups displayed a significant (*p* < 0.001) increase in PDW% compared with the healthy control group (Fig. 3). Regarding PLT indices, the diabetic heart failure group displayed a significant (*p* < 0.05) increase in the PLT count compared with the healthy control group (Table 2), whereas all CVD patient groups revealed a marked increase in MPV values, except for the diabetic myocardial infarction group, compared with the healthy control group. Meanwhile, the diabetic CVD patient groups showed a marked (*p* < 0.05) increase in PCT% compared with the healthy control and nondiabetic groups (Fig. 4).

**Table 2:** Hematological Profile of Healthy Controls and Diabetic and Nondiabetic CVD Groups

Group	Healthy Controls	Non Diabetic myocardial infraction	Diabetic myocardial infraction	Non Diabetic Heart failure	Diabetic Heart failure
Parameter	(n=30)	(n=30)	(n=30)	(n=30)	(n=30)
Hb (g/dl)	12.7±0.9	12.8±0.3 N.S.	13.5±0.3N.S.	11.0±0.3***	11.6±0.3*
WBCS (10³/µl)	5.9 ±0.3	8.7±0.6*	12.4±1.2***#	9.1±1.0*	7.2±0.6N.S.
RBCS (M/µl)	4.7±0.1	4.6±0.1N.S.	5.0±0.1 N.S.	4.0±0.1***	4.2±0.1*
Platelets (10³/µl)	231.5±9.0	249.5±13.3 N.S.	247.7±9.9 N.S.	266.8±15.1N.S.	273.7±18.3*
HCT%	38.5±0.7	39.5±0.9 N.S.	42.4±1.0	34.0±0.9**	35.5±1.1*
MCV(fl)	82.2±1.0	86.9±1.4*	84.9±0.7 N.S.	85.2±1.4 N.S.	85.8±0. 9 *
MCH (pg)	27.3±0.3	28.2±0.6 N.S.	27.1±0.4 N.S.	27.4±0.5 N.S.	28.0±1.7 N.S.
MCHC(g/dl)	33.2±0.3	32.5±0.4 N.S.	31.9±0.3N.S.	32.3±0.3 N.S.	32.7±0.4N.S.
N%	57.9±2.4	70.4±1.9***	73.9±2.0***	65.2±2.4*	65.6±2.8*
L%	37.8±2.5	20.2±1.7***	20.2±1.7***	28.7±2.5*	29.1±2.8*
M%	3.1±0.2	3.5±0.4 N.S.	4.0±0.4 N.S.	4.1±0.6N.S.	3.7±0.5 N.S.

Mean values and SEM are represented. A non-parametric Kruskal–Wallis test followed by the post hoc test using a pairwise multiple-comparative analysis was used to determine the statistical difference between groups, number of individuals = 150. *p < 0.05, **p < 0.01, and ***p < 0.001 compared with the healthy control group and with #*p* < 0.05 compared with the respective nondiabetic groups

*NS* not significant, *Hb* hemoglobin, *WBCs* white blood cells, *RBCs* red blood cells, *HCT* hematocrit, *MCV* mean corpuscular volume, *MCH* mean corpuscular hemoglobin, *MCHC* mean corpuscular hemoglobin concentration, *N* neutrophil, *L* lymphocytes, and *M* monocytes

The WBC count increased significantly (*p* < 0.001) in all CVD patient groups, except for the diabetic heart failure group, compared with that in the healthy control group (Table 2). However, the neutrophil count exhibited a significant increase in all CVD patient groups compared with that in the healthy control group. Conversely, the lymphocyte count showed a remarkable decline in all CVD patient groups compared with that in the healthy control group (Table 2). Concerning PLT-to-lymphocyte (P/L) and neutrophil-to-lymphocyte (N/L) ratios, the results showed a noticeable increase in all CVD patient groups compared with those in the healthy control group. Additionally, the P/L and N/L ratios exhibited remarkable increases in the diabetic CVD patient groups compared with those in the nondiabetic CVD patient groups. However, the lymphocyte-to-monocyte (L/M) ratio revealed a significant decrease in both myocardial infarction patient groups compared with that in the healthy control group, with a remarkably high decrease in the diabetic myocardial infarction patient group (Fig. 4).

In the diabetic CVD groups, HbA1c% displayed a significant positive correlation with IL-6, IL-18, IL-38, MDA, and PDW%, whereas a negative correlation was detected with IL-35 and SOD. In addition, CK-MB exhibited a significant positive correlation with IL-6, IL-18, IL-38, MDA, PDW%, and P/L and N/L ratios, whereas a significant negative correlation was observed with IL-35 and SOD. Moreover, the anti-atherogenic index demonstrated a significant positive correlation with IL-36 and SOD, whereas a significant negative correlation was observed with IL-6, IL-18, IL-38, MDA, PDW%, PCT%, and P/L and N/L% ratios (Table 3).

**Table 3:** Correlation of HbAIc%, CK-MB, and Anti-Atherogenic with IL-38, IL-6, IL-38, IL-36, MDA, SOD, PDW, PCT, P/L Ratio, and N/L Ratio in Diabetic CVD Groups

*P* value	*r* value	Correlated parameters
< 0.01	0.226	HbA1c %/IL-6
< 0.01	0.225	HbA1c %/IL-18
< 0.01	− 0.346	HbA1c %/IL-35
< 0.01	0.258	HbA1c %/IL-38
< 0.01	0.204	HbA1c %/MDA
< 0.001	− 0.428	HbA1c %/SOD
< 0.01	0.278	HbA1c %/PDW%
> 0.05	0.084	HbA1c %/PCT
> 0.05	0.067	HbA1c %/P/L
> 0.05	0.021	HbA1c %/N/L
< 0.01	0.492	CK-MB/IL-6
< 0.001	0.593	CK-MB/IL-18
< 0.01	− 0.346	CK-MB/IL-35
< 0.001	0.539	CK-MB/IL-38
< 0.01	0.580	CK-MB/MDA
< 0.001	− 0.368	CK-MB/SOD
< 0.01	0.496	CK-MB/PDW%
> 0.05	0.190	CK-MB/PCT%
< 0.001	0.484	CK-MB/P/L
< 0.01	0.447	CK-MB/N/L
< 0.01	− 0.572	Anti-atherogenic/IL-6
< 0.001	− 0.571	Anti-atherogenic/IL-18
< 0.01	0.481	Anti-atherogenic/IL-35
< 0.01	− 0.565	Anti-atherogenic/IL-38
< 0.001	− 0.620	Anti-atherogenic/MDA
< 0.01	0.560	Anti-atherogenic/SOD
< 0.01	− 0.547	Anti-atherogenic/PDW%
< 0.01	− 0.220	Anti-atherogenic/PCT%
< 0.01	− 0.333	Anti-atherogenic/P/L
< 0.01	− 0.256	Anti-atherogenic/N/L

The correlations between HbAIc%, CK-MB, and anti-atherogenic with IL-38, IL-6, IL-38, IL-36, MDA, SOD, PDW, PCT, P/L ratio, and N/L ratio were established by Spearman’s correlation analysis. r, Spearman’s correlation coefficient

## DISCUSSION

After controlling for inflammatory markers and classical risk factors, patients with diabetes had almost twice the risk of cardiovascular mortality compared with those without diabetes [1]. Furthermore, the increase in the number of patients with T2DM highlights the necessity of early CVD detection in patients with diabetes [6]. Thus, this study was anticipated to explore the interplay of DM-induced oxidative stress and inflammation with hematological abnormalities in CVD risk.

In our study, the myocardial infarction and heart failure groups exhibited a significant increase in serum cholesterol, triglyceride, LDL, vLDL-c levels, and risks factors 1 and 2, compared with the healthy control group. However, HDL levels and the anti-atherogenic index showed a notable reduction in the diabetic CVD groups compared with those in the nondiabetic CVD and healthy control groups. High glucose levels and dyslipidemia are complicated in atherosclerosis pathogenesis that eventually leads to CVD deaths. Chronic inflammation is one of the leading causes of atherosclerosis development [17]. The increase in serum CK-MB, troponin I, and lipid profile markers, including risks factors 1 and 2, confirmed the cardiac dysfunction of patients with diabetes, which leads to exploring diabetes cardiomyopathy complications.

In this study, MDA displayed a significant increase in the CVD patient groups with a marked elevation in the diabetic groups compared with the healthy control and nondiabetic groups. At the same time, SOD levels exhibited a remarkable decline in all CVD groups with a significant decrease in diabetic patients with CVD compared with those in the healthy control nondiabetic groups. Our results revealed a significant correlation among both MDA and SOD and HbA1c, CK-MB, and the anti-atherogenic index, indicating that dyslipidemia- and hyperglycemia-associated oxidative stress may induce the development of diabetic cardiomyopathy complications. These results are consistent with the study of Fathelbab *et al*. [18] in which it was observed that MDA levels were significantly increased in diabetic patients with CVD, and SOD levels in the diabetic CVD group were significantly reduced compared with those in the control group, indicating the role of oxidative stress-mediated tissue injury in diabetic patients with CVD. Moreover, a marked reduction in all antioxidant enzymes was observed, including SOD, in patients with metabolic syndrome (patients with dyslipidemia, hypertension, and T2DM) compared with the healthy controls [19]. Oxidative stress in heart failure occurs as a consequence of the excessive ROS production that can enhance lipid peroxidation and oxidize proteins to inactive states and cause DNA damage [20]. In addition, high ROS production may trigger maladaptive signaling pathways, which results in cell death and promotes abnormal cardiac remodeling, ultimately leading to diabetic cardiomyopathy-related functional abnormalities [21]. SOD is a member of the metalloproteinase family, and its overproduction is related to hypertension, diabetes, and CVD [22]. Additionally, SOD decline was associated with endothelial dysfunctions and hypertension [19]. When acute myocardial infarction occurs, it almost reduces SOD’s ability to scavenge free radicals. Increased MDA levels, followed by ROS accumulation, resulted in acute myocardial infarction [23]. Moreover, low antioxidant enzyme levels led to excessive insulin resistance that enhanced stress-related pathways, leading to diabetic cardiovascular events [24].

CVD is an inflammatory condition, whereas CRP is an acute-phase protein. Notably, clinical studies have demonstrated that CRP is a predictor of CVD [25] and associated complications [26]. The current study showed that all CVD groups (myocardial infarction and heart failure) exhibited higher CRP levels compared with the healthy controls. Moreover, CVD groups with diabetes had higher CRP levels than the corresponding normoglycemic groups. Furthermore, our results suggest a strong correlation between inflammatory cytokines and oxidative biomarkers in diabetic CVD groups. Our results are consistent with the results of previous studies, which declared that CRP is a novel marker for predicting CVDs in patients with diabetes mellitus [27]. Thus, our findings indicate that diabetes may worsen inflammatory responses and oxidative stress and could contribute to adverse outcomes in CVD.

Notably, the current data found a strong correlation between pro-inflammatory cytokines (IL-6, IL-18, and IL-38) and HbA1c, CK-MB, and anti-atherogenic index biomarkers. Additionally, the results showed that IL-6, IL-18, and IL-38 mRNA expressions exhibited a significant upregulation in the diabetic and nondiabetic CVD groups compared with those in the healthy control group, with a highly significant increase in diabetic patients with CVD. Cytokines, TNF-α, IL-1β, and IL-6, were found to be elevated in patients with heart failure, and their higher levels appear to be directly related to LVEF dysfunction [28]. Myocardial damage, heart failure, and mortality are linked to high levels of circulating IL-6 during and immediately after an acute myocardial infarction [29]. IL-6 levels in patients with left ventricular diastolic dysfunction (LVDD) were found to be substantially linked with the levels of fibrotic parameters, suggesting that IL-6 may play a role in increasing myocardial fibrosis and LV remodeling, finally leading to LVDD [30].

IL-18 is a pro-inflammatory cytokine that is predominantly produced by macrophages and binds to its receptor on the membranes of endothelial cells, lymphocytes, and smooth muscle cells to cause the production of interferon gamma, endothelial dysfunction, and plaque instability [31]. Our results are consistent with the results of Xiao *et al*. [32] in which it was revealed that increased IL-18 levels have been related to an increased risk of CVD. Several studies found that IL-18 was considerably higher in the plasma of patients who had coronary events [33], which agrees with our results. Notably, continued IL-18 suppression by IL-18-binding protein leads to cardiac fibrosis reduction and NF-κB phosphorylation, diastolic function improvement, electrical remodeling normalization, and IL-18-mediated ventricular tachycardia reduction in mice [34]. The IL-38 mRNA expression was upregulated in the mouse heart following myocardial infarction and exhibited a decrease in dendritic cell–mediated immune response [35]. Consequently, targeting IL-38 may hold new therapeutic approaches in the treatment of patients with myocardial infarction.

Moreover, our data demonstrated a correlation among IL-35 and HbA1c, CK-MB, and anti-atherogenic index biomarkers; it may be a novel therapeutic target in patients at risk for diabetic CVD. IL-35 has been linked to several cardiovascular disorders, including atherosclerosis and viral myocarditis [36]. IL-35 reduces doxorubicin-induced heart damage by increasing STAT3 signaling, lowering oxidative stress, and blocking mitochondrial-related apoptotic pathways [37]. The abovementioned studies are consistent with our study, showing a significant decrease in IL-35 levels in the CVD patient groups compared with those in the healthy control group. IL-35 can reduce myocardial ischemia/reperfusion (I/R) dysfunction by reducing mtROS. Furthermore, it was believed that IL-35 protects cardiomyocytes by inhibiting apoptosis, thereby minimizing cardiac I/R injury [38].

Furthermore, anemia is related to microvascular complications, diabetic nephropathy, neuropathy, and CVD [39]. HB content, RBCs count, and HCT% exhibited a significant decrease in CVD groups compared with healthy controls. Moreover, RBCs of a patient with diabetes combined more willingly, thereby augmenting whole blood viscosity and harming the microcirculation, finally leading to microangiopathy [40]. Moreover, all CVD patient groups revealed a significant increase in PDW% compared with the healthy control group. A high RDW is a disease severity metric associated with a variety of adverse outcomes, including CV and non-CV death, in patients with T2DM who have recently had an acute coronary syndrome [41]. Despite the completely unclear mechanisms between the RDW and adverse health consequences, it was recommended that it might be associated with the increase in ROS and pro-inflammatory cytokine levels [42].

PLTs are essential for maintaining normal homeostasis, and MPV is the indicator of their function. PLTs play a substantial role in atherosclerosis development and acute thrombotic cardiovascular event progression [43]. Our findings of higher MPV among patients with T2DM are consistent with those of several studies [44] that reported that the diabetic CVD group showed a significant increase in MPV compared with the healthy group. Elevated MPV has been seen in diabetic patients with retinopathy, nephropathy, and coronary heart disease; it represents alterations in PLT stimulation or PLT production rate [45]. PDW is a specific PLT reactivity biomarker that can help predict CVD [46], which is consistent with our study that showed that the CVD group showed a significant increase in PDW compared with the healthy control group. In our study, the CVD groups showed a significantly increased PCT% compared with the healthy control group. PLTs release various mediators, including thromboxanes, which can cause inflammation; increased PCT levels at the time of admission have been linked to poor long-term outcomes in patients with myocardial infarction [47].

WBCs are a biological systemic inflammation indicator, and an increased WBC count is linked to an increased risk of CAD, mortality rate, and stroke [48]. In this study, the CVD groups demonstrated a significant elevation in the neutrophil count compared with the healthy control group. The current results revealed that CK-MB and the anti-atherogenic index exhibited a positive correlation with the P/L and N/L ratios. Neutrophils are leukocytes that serve as the first line of defense against pathogens and damages caused by inflammation. The increase in neutrophil blood count has been linked to the severity of coronary damage and the prevalence of heart failure [49]. Lymphocytes reflect a calm and controlled immune response that causes less cardiac damage; lymphocyte levels decrease as apoptosis increases. In individuals with chronic heart failure, a low blood lymphocyte count has been linked to worse cardiovascular outcomes [50].

Recently, PDW, MPV, and N/L and P/L ratios were identified as microvascular diabetic issue indicators and announced as novel inflammatory biomarkers in cardiac diseases. The results of the previous studies [51, 52] are consistent with the results of our study, revealing that the N/L ratio had a significant increase in the CVD groups compared with that in the healthy control group. The N/L ratio, a simple, inexpensive, and innovative inflammatory biomarker, was shown to be higher in patients with diabetes and was related to poor glycemic control and may be prognostic predictors in cardiovascular events [53]. In patients with heart failure, neutrophilia, lymphopenia, and a higher N/L ratio have been linked to heart failure severity [54].

Additionally, our results showed that the N/L, L/M, and P/L ratios beside MPV, PDW, and PCT% were considered novel inflammatory biomarkers, confirming that the impaired hematological indices are one of the diagnostic markers in the development of diabetic cardiomyopathy. Also, the P/L and N/L ratios exhibited remarkable elevations in the diabetic CVD patient groups compared with those in the nondiabetic CVD patient groups. The P/L ratio is significantly and independently linked with the occurrence of in-hospital severe adverse cardiovascular events as a novel inflammatory marker. In patients with acute myocardial infarction, there was additional evidence of a link between the P/L ratio and long-term prognoses [55]. Additionally, the combination of the N/L and P/L ratios had both short- and long-term predictive significance [56]. These previous studies [55, 56] agree with our study, which reported a significant increase in the P/L ratio in the CVD groups compared with that in the healthy control group. Moreover, our findings revealed that the CVD groups exhibited a significant decrease in the L/M ratio compared with the healthy control group. These findings were consistent with those of [57] which reported that the L/M ratio was also linked to the prognosis of individuals with myocardial infarction, heart failure, and stable CAD. Notably, a lower L/M ratio is linked to a higher frequency of unstable angina pectoris or myocardial infarction in patients with CVD. Furthermore, the L/M ratio is an independent indicator of heart failure re-hospitalization in patients with CVD [58]. The study had some limitations, however, including the sample size and separation of the results based on age, sex, and disease duration. Moreover, this study lacks data on physical activity, drug use, and the serum protein levels of the tested cytokines.

In conclusion, our findings suggest a possible association of an oxidative stress represented by excess of MDA production and reduced SOD activity, and inflammatory state represented by increased mRNA expressions of the pro-inflammatory cytokines, IL-6, IL-18, and IL-38 and reduced expression of anti-inflammatory IL-35, and the progression of the diabetic cardiovascular diseases. Moreover, impaired hematological indices, including RDW, PLT, PDW%, PDW, PCT%, and N/L, L/M, and P/L ratios, were associated with the development of diabetic cardiomyopathy. Hematological indices are particularly sensitive to systemic inflammatory changes and may be novel markers for predict the progression of diabetic CVD.

## Data Availability

All data generated or analyzed during this study are included in the article.
